# Sex Differences in Mesenchymal Stem Cell Therapy With Gelatin-Based Microribbon Hydrogels in a Murine Long Bone Critical-Size Defect Model

**DOI:** 10.3389/fbioe.2021.755964

**Published:** 2021-10-19

**Authors:** Masaya Ueno, Ning Zhang, Hirohito Hirata, Danial Barati, Takeshi Utsunomiya, Huaishuang Shen, Tzuhua Lin, Masahiro Maruyama, Ejun Huang, Zhenyu Yao, Joy Y. Wu, Stefan Zwingenberger, Fan Yang, Stuart B. Goodman

**Affiliations:** ^1^ Department of Orthopaedic Surgery, Stanford University, Stanford, CA, United States; ^2^ Department of Orthopaedic Surgery, Faculty of Medicine, Saga University, Saga, Japan; ^3^ Department of Medicine, Stanford University, Stanford, CA, United States; ^4^ University Center for Orthopaedics, Traumatology, and Plastic Surgery, University Hospital Carl Gustav Carus at Technische Universität Dresden, Dresden, Germany; ^5^ Department of Bioengineering, Stanford University, Stanford, CA, United States

**Keywords:** sex differences, bone healing, mesenchymal stem cell, microribbon hydrogel, interleukin-4

## Abstract

Mesenchymal stem cell (MSC)-based therapy and novel biomaterials are promising strategies for healing of long bone critical size defects. Interleukin-4 (IL-4) over-expressing MSCs within a gelatin microribbon (µRB) scaffold was previously shown to enhance the bridging of bone within a critical size femoral bone defect in male Balb/c mice. Whether sex differences affect the healing of this bone defect in conjunction with different treatments is unknown. In this study, we generated 2-mm critical-sized femoral diaphyseal bone defects in 10–12-week-old female and male Balb/c mice. Scaffolds without cells and with unmodified MSCs were implanted immediately after the primary surgery that created the bone defect; scaffolds with IL-4 over-expressing MSCs were implanted 3 days after the primary surgery, to avoid the adverse effects of IL-4 on the initial inflammatory phase of fracture healing. Mice were euthanized 6 weeks after the primary surgery and femurs were collected. MicroCT (µCT), histochemical and immunohistochemical analyses were subsequently performed of the defect site. µRB scaffolds with IL-4 over-expressing MSCs enhanced bone healing in both female and male mice. Male mice showed higher measures of bone bridging and increased alkaline phosphatase (ALP) positive areas, total macrophages and M2 macrophages compared with female mice after receiving scaffolds with IL-4 over-expressing MSCs. Female mice showed higher Tartrate-Resistant Acid Phosphatase (TRAP) positive osteoclast numbers compared with male mice. These results demonstrated that sex differences should be considered during the application of MSC-based studies of bone healing.

## Introduction

Sex is one of the critical factors that influences the types and frequencies of diseases in the musculoskeletal system. For example, osteoporosis is more prevalent in women than in men (Cawthon, 2011). Osteoporosis affects up to 30% of women but only 12% of men at some point in their lives (Ralston and de Crombrugghe, 2006). The risk of fracture is also higher in women than in men (Cawthon, 2011); women show a greater incidence of stress fractures early in life and fragility fractures later in life (Cummings et al., 2002; Beck et al., 2000). In a clinical study, female sex was found to be associated with a reduced fracture union rate (Chang et al., 2006), and was identified as a major risk factor for compromised bone healing in several clinical studies (David et al., 2006; Li et al., 2006; Parker et al., 2007). Thus, it is prudent to consider the premise that the treatment of musculoskeletal disorders including bone healing should be tailored to the patient’s sex.

Mesenchymal stem cell (MSC) therapies have shown great potential to facilitate bone healing (Shi et al., 2010; Liu et al., 2014a; Sui et al., 2019; Zhang et al., 2021). MSCs are one of the most investigated adult stem cell populations and are involved in the continuous maintenance and repair of many tissue types (Kern et al., 2006; Jin et al., 2013). For example, systemic transplantation of MSCs has shown remarkable efficacy in preventing and treating estrogen deficiency-induced bone loss in pre-clinical studies (Cho et al., 2009; Lee et al., 2011; An et al., 2013; Liu et al., 2014b; Sui et al., 2017).

We have encapsulated MSCs within gelatin microribbon (µRB)-based scaffolds to enhance the efficacy of mesenchymal stem cell delivery to a bone defect site with high cell survival and adequate supply of nutrients and provide primary structural support (Han et al., 2016; Conrad et al., 2018). μRB building blocks can be homogeneously mixed with cells with high cell viability and can inter-crosslink into macroporous scaffolds (Han et al., 2013). Macroporous gelatin μRB-based scaffolds increased cartilage formation in a murine subcutaneous implantation model (Rogan et al., 2020). In a murine cranial defect, μRB-based scaffolds significantly improved stem cell survival, vascular ingrowth, and accelerated bone formation (Han et al., 2016).

It is recognized that inflammation and the innate immune system including macrophages play crucial roles in the differentiation and activation of MSCs, and are essential for normal bone growth, repair, and homeostasis (Chow et al., 2019; Maruyama et al., 2020). Acute injury damages bone, the local soft tissues, and the neurovascular system, leading to the production of a pro-inflammatory microenvironment. This inflammatory milieu is the first stage of bone healing and determines the delicate balance between bone formation and bone degradation (Loi et al., 2016a). The inflammatory cascade polarizes macrophages to a proinflammatory M1 phenotype; M1 macrophages produce cytokines and chemokines that recruit MSCs, vascular progenitors and other cells, and activates or licenses MSCs to a new state facilitating resolution, re-vascularization, reconstruction, and homeostasis. As the acute inflammatory response subsides, M2 anti-inflammatory macrophages further stimulate tissue regeneration.

IL-4 is an anti-inflammatory cytokine; when IL-4 was added in monoculture of mouse MSCs acutely, IL-4 decreased cell proliferation and osteogenic differentiation (Lin et al., 2017a; Zhang et al., 2021). However, IL-4 stimulates the polarization of macrophages from an M1 to an M2 phenotype (Shapouri-Moghaddam et al., 2018), and crosstalk between MSCs and macrophages is critical for successful bone healing (Pajarinen et al., 2019). Interestingly, IL-4 over-expressing MSCs enhanced osteogenesis in MSC-macrophage cocultures if the IL-4 was added after 48–72 h (Lin et al., 2019). To aid in this reparative response, we developed IL-4 over-expressing MSCs using lentiviral vectors. IL-4 over-expressing MSCs within μRB scaffolds, delivered locally 3 days after the acute injury, enhanced bone formation using a long bone defect in young, male mice (Ueno et al., 2020) in an established femoral diaphyseal critical size bone defect model (Zwingenberger et al., 2013). Whether this biological approach is comparable in female mice having a different hormonal profile is unknown.

In the current study, we compare the healing potential of IL-4 over-expressing MSCs in male and female mice using an established murine long bone critical size defect model. We evaluated the healing defect using comprehensive histologic and immunohistochemical analyses, and µCT of the bone defect site.

## Materials and Methods

### Isolation and Manipulation of MSCs

Bone marrow derived MSCs from each sex were isolated according to a previously published method (Peister et al., 2004; Lin et al., 2015). Briefly, we collected bone marrow from both femurs and tibias of 8–10-week-old BALB/c male and female mice. Then bone marrow was carefully suspended and filtered through a 70 μm strainer, spun down and resuspended in alpha-minimal essential medium (α-MEM, Thermo Fisher Scientific, Waltham, MA, United States) supplied with 10% fetal bovine serum (FBS, Invitrogen, Carlsbad, CA, United States) and antibiotic antimycotic solution (100 units of penicillin, 100 μg of streptomycin and 0.25 μg of Amphotericin B/ml; Hyclone, Thermo Fisher Scientific, Waltham, MA, United States). The unattached cells were removed by replacing the culture media after 24 h and defined as passage 1. The immunophenotype of isolated MSCs according to International Society for Cell Therapy (ISCR) (Dominici et al., 2006) (CD105+ /CD73+ /CD90.2+ /Scal + CD45−/ CD34−CD11b−) was characterized by flow cytometry (LSR II, Stanford Shared FACS Facility, Stanford, CA, United States) at passage 4. MSCs between passage four to eight were used in the current study. Based on our protocol (Ueno et al., 2020), we produced genetically modified MSCs that over-express IL-4 by infecting MSCs with the lentiviral vector carrying murine IL-4 gene. Briefly, the lentivirus vectors were generated in HEK293T cells by co-transfecting with the transfer plasmid (pCDH-CMV-mIL-4-EF1-copGFP), packaged plasmid (psPAX2), and enveloped plasmid (pMD2G VSVG) using a calcium phosphate transfection kit (Takara Bio United States Inc., Mountain View, CA, United States) with 25 mmol/L chloroquine. We then collected the supernatants of the culture media 48 h after the transfection, and the cellular debris was removed by centrifugation at 4,000 g for 20 min. The virus was mixed in MSC culture medium supplemented with 6 μg/ml of polybrene (Sigma Aldrich, St. Louis, MO, United States) with the multiplicity of infection 100 for MSC infection (Ricks et al., 2008; Lin et al., 2017b; Zhang et al., 2021). ELISA kits for mouse IL-4 (R&D system, Minneapolis, MN, United States) were used to quantify IL-4 expression by the infected MSCs (IL-4 MSCs) or non-infected MSCs (MSCs) for 24 h culture. The manufacturers’ protocols were carefully followed. The optical densities were determined using SpectraMax M2e Microplate Readers (Molecular Devices, San Jose, CA, United States) set at 450 nm with wavelength correction set to 540 nm.

### Gelatin μRB-Based Scaffold

**FIGURE 1:**
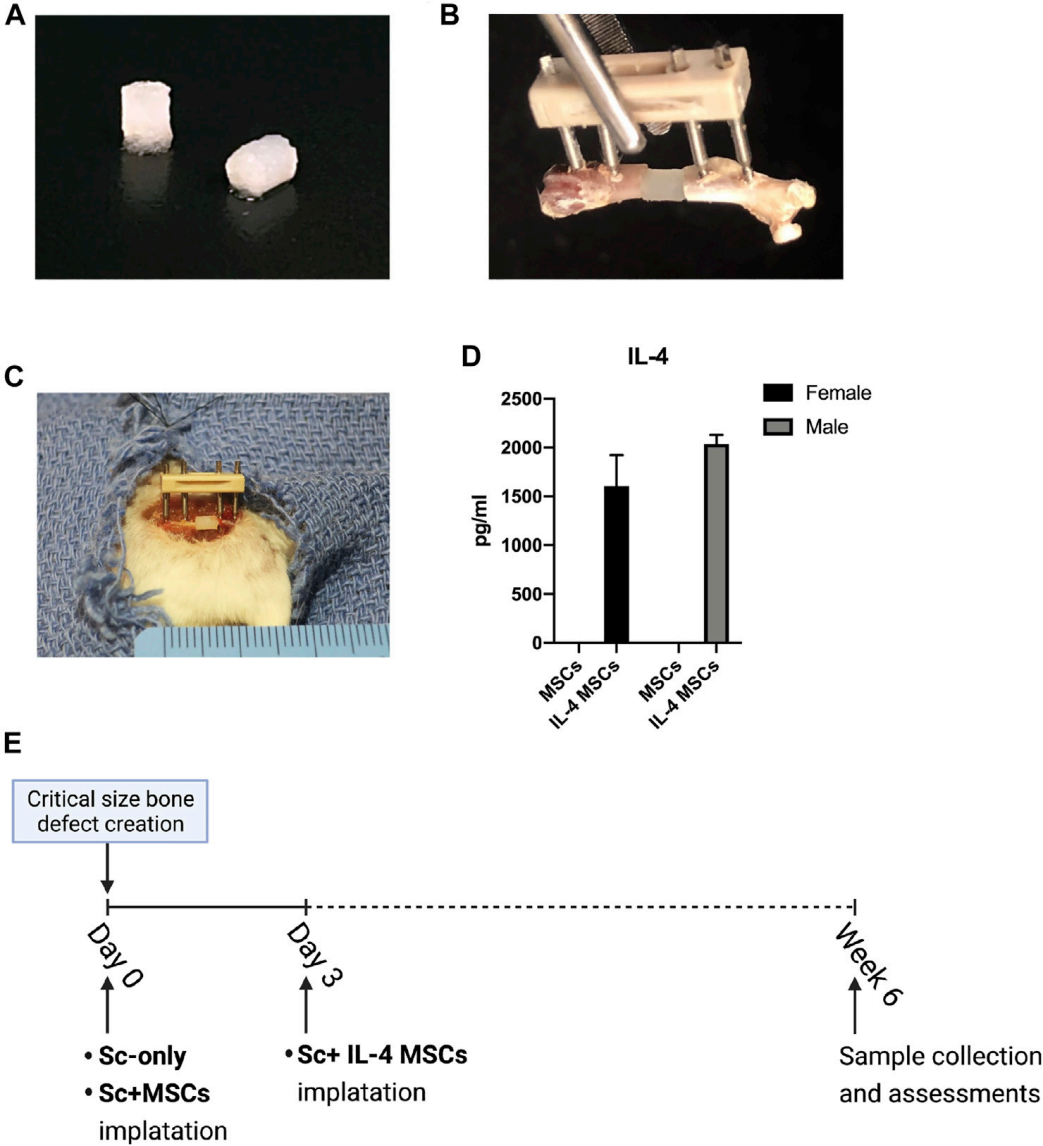
Animal model and surgical procedure. **(A)** MSC-seeded μRBs scaffolds. **(B)** A model of the external fixator and μRBs. **(C)** Implantation of a μRBs scaffold in a mouse femur critical size bone defect with an external fixator. **(D)** IL-4 secreting levels detected by ELISA in the culture media of IL-4 MSCs and MSCs for 24 h (2039.01 ± 90.73 pg/ml in male IL-4 MSCs and 1607.25 ± 313.58 pg/ml in female IL-4 MSCs. The IL-4 concentration could not be detected by ELISA in male and female MSC alone groups). **(E)** Surgical procedure. The IL-4 overexpressing MSCs (IL-4 MSCs) were added day 3 after creation of the surgical defect, whereas in the other groups, namely scaffold only (Sc-only), scaffold with unmodified MSCs (Sc + MSCs), were added immediately after the surgery (day 0).

The fabrication of gelatin μRBs (Figure 1A) using a wet spinning process was conducted according to a previous report (Conrad et al., 2018). Briefly, gelatin was stirred in dimethyl sulfoxide (20 wt%) at 60°C for 18 h at 60 rpm to form a viscous solution. Then the gelatin solution was transferred to a 60 ml syringe and ejected using a syringe pump set to 5 ml/h into ethanol located 1.8 m under the syringe being stirred at 500 rpm. The precipitated gelatin microfiber was transferred to acetone for 3 h to dry and form μRBs. The μRBs were chopped to short length using a homogenizer after transferred back to ethanol. The μRBs were transferred to methanol containing methacrylic acid N-hydroxysuccinimide ester (15 wt%) and stirred for 18 h at room temperature to functionalize them. Next, the μRBs were transferred to fresh methanol containing glutaraldehyde (0.1 wt%) and stirred vigorously for 18 h at room temperature. The glutaraldehyde was neutralized by adding L-lysine hydrochloride (1% in 200 ml PBS) and stirring for 2 h. The product was washed eight times using PBS and three times using deionized water to remove the reagents, thereafter, the product was freeze-dried and stored at −20°C.

To fabricate scaffolds, the μRBs were rehydrated using PBS containing 0.05% LAP photo-initiator. After incubation for 1 h at 37°C, the μRBs were gently mixed with trypsinized MSCs suspended in PBS. The cell concentrations were 10 million cells/mL. The μRBs containing cells were filled to 2 mm diameter cylindrical mold and exposed to ultraviolet light (365 nm, 2 mW/cm^2^) for 4 min to produce macroporous scaffolds. The scaffolds were then gently pushed out from the mold and kept in PBS for further applications. Cell properties after encapsulation into the same scaffold prior to placement of this construct into the bone defect, as well as cell viability in the scaffolds after placement into a defect site in a mouse cranial defect model were tested and reported previously (Han et al., 2016). Bioluminescence imaging (BLI) showed that 81.1 ± 21.3% of stem cells in the µRB scaffold were alive at day 1, compared to 26.6 ± 10.1% in the hydrogel scaffold group. After 6 weeks, almost 40% of stem cell still showed BLI positive signals.

### Animals

Stanford’s Administrative Panel on Laboratory Animal Care (APLAC) approved this animal experiment protocol (APLAC 26905). Guidelines for the Care and Use of Laboratory Animals were followed in all aspects of the current project. Male and female Balb/c mice (Jackson Laboratory, Bar Harbor, ME) between 10 and 12 weeks old were used. These mice were kept on a 12-h light-and-dark cycle, and they were able to access a standard diet with food and water ad libitum.

### Surgical Procedure and Postoperative Care

The surgery was conducted on mice who were given preoperative analgesia by subcutaneously injection of 0.1 mg/kg of buprenorphine. Mice were anesthetized using inhalation anesthesia with isoflurane in 100% oxygen at a flow of 1 L/min on a warm surgery station for small animals during the surgical procedures. The surgery was conducted by at least two experienced surgeons in each case, and one non-operative surgeon assistant. A 2-mm critical-sized diaphyseal bone defect was made in the femur, as previously described (Figures 1B,C) (Zwingenberger et al., 2013). Briefly, we approached the right femur *via* a longitudinal lateral incision, then implanted the femoral external fixation device (MouseExFix, RISystem AG, Landquart, Switzerland) onto the right femur. Then, a special jig was used to generate a 2 mm critical-size bone defect in the midshaft of the femur using a Gigli saw (Zwingenberger et al., 2013). There were three groups for each sex: μRB scaffold without any implantation of cells (Sc-only group), μRB scaffold with unaltered MSCs (Sc + MSCs group), and μRB scaffold with IL-4 over-expressing MSCs (Sc + IL-4-MSCs group). Mice were implanted with MSCs which were isolated from the same sex. Male recipient mice received male donor MSCs or IL-4 MSCs, whereas female recipient mice received female donor MSCs or IL-4 MSCs.

The surgical procedure and scaffold implantation for Sc-only group and Sc + MSCs group were performed at time zero. Since the previous *in vitro* study demonstrated that administration of the anti-inflammatory cytokine IL-4 during the first 48 h of culture significantly mitigated acute inflammation, decreased cell proliferation and downregulated oncostatin M which is recognized to enhance osteogenesis (Guihard et al., 2012; Loi et al., 2016b), the scaffolds with IL-4 over-expressing MSCs were implanted 3 days after the primary surgery (Figure 1D). We closed the surgical incisions with 5–0 Ethilon sutures and injected BuprenorphineSR (0.1 mg/kg) subcutaneously for analgesia after surgery.

### Micro-Computational Tomography and Radiographic Analysis

**FIGURE 2:**
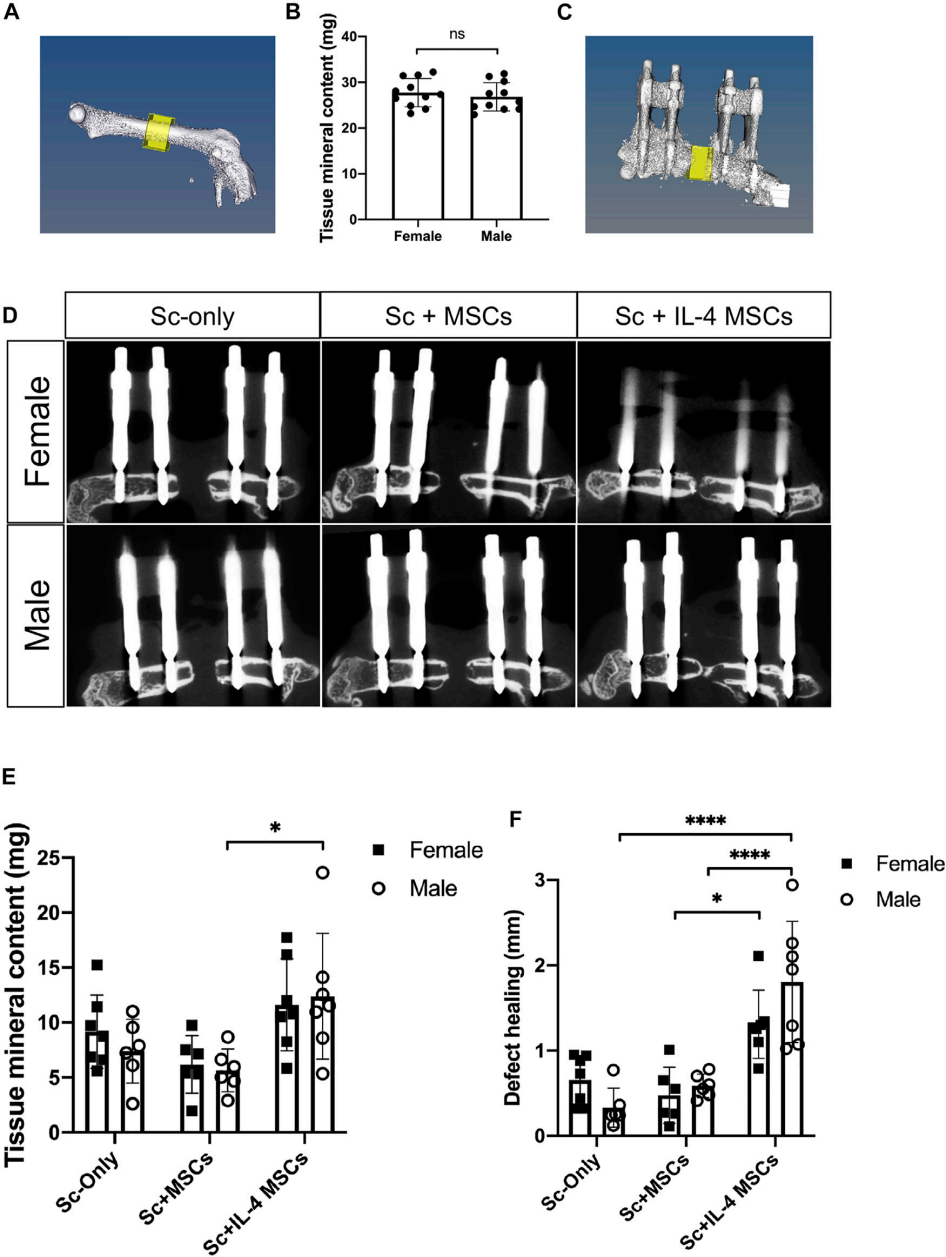
µCT -based reconstructions of the femurs in male and female mice. **(A)** µCT -based reconstructions of the healthy side of femur and the region of interest. **(B)** Tissue mineral content (mg) of the region of interest. female, *n* = 16; male, *n* = 16. IL-4 MSCs in μRBs decreased the sizes of bone defects in both sexes. **(C)** µCT -based reconstructions of the initial defect area and the region of interest. **(D)** µCT -based reconstructions of femurs and defect areas of three groups. **(E)** Tissue mineral content (mg) of the bone defect area and **(F)** defect healing at 6 weeks (mm) calculated by the µCT. Female Sc-only, *n* = 7; female Sc + MSCs, *n* = 6; female Sc + IL-4 MSCs, *n* = 7; male Sc-only, *n* = 6; male Sc + MSCs, *n* = 6; male Sc + IL-4 MSCs, *n* = 7. *: 0.01 ≤ *p* < 0.05, ****: *p* < 0.0001.

6 weeks after the primary surgery, mice were euthanized by exposure to CO_2_ followed by cervical dislocation. Both lower limbs from each animal were collected. µCT scans were performed using a TriFoileXplore CT 120 (TriFoil Imaging, Chatsworth, CA) with 50 μm resolution (Ueno et al., 2020; Utsunomiya et al., 2021a; Utsunomiya et al., 2021b). In the left femur (the healthy side), a 3 × 3 × 2 mm rectangular region in the center of femur was scanned (Figure 2A). For the right femur containing the 2 mm critical-size bone defect surgery, we measured the original length of defect based on the µCT images case by case to set the size of ROI (3 mm × 3 mm × original length) (Figure 2C). Final length of the defect was also measured case by case. We calculated the tissue mineral content of the newly formed bone from the original bone defect area. The length of the bone defect healing was also calculated by this formula: defect healing (mm) = original length (mm)—final length (mm) (Figure 2).

### Histologic and Immunohistochemical Analysis

The tissue samples were fixed in 4% paraformaldehyde overnight after the µCT scans, then decalcified in 0.5 M ethylenediaminetetraacetic acid (EDTA) for 2 weeks, then embedded in optimal cutting temperature compound (OCT) and frozen at −80°C. Embedded samples were cut into 10 μm-thick sections. Hematoxylin and Eosin (H&E) staining was performed for histological analysis. To evaluate the status of bone healing, the contents of the defect were graded according to the scoring system of Huo et al., (1991) (Table.1). 1-Step NBT/BCIP Substrate Solution (Thermo Fisher Scientific Rockford, IL) was used for Alkaline phosphatase (ALP) staining. After staining with 1-Step NBT/BCIP Substrate Solution, the ALP-positive area based on the entire area of the scaffold was calculated using the image analysis software program ImageJ (National Institutes of Health, Bethesda, MD, United States) (Schneider et al., 2012). Osteoclast-like cells were determined using a leukocyte tartrate resistant acid phosphatase (TRAP) staining kit (Sigma Aldrich, St. Louis, MO, United States) and counted as TRAP positive multi-nucleated cells located in the bone defect area. To identify the macrophages, the sections were blocked by 5% BSA buffer for 30 min at room temperature, followed by 1 h primary and secondary antibody incubation at room temperature. Macrophages were stained by rat anti-CD11b antibody (Abcam, Cambridge, MA, United States) followed by Alexa Fluor® 647 conjugated donkey anti-rat IgG (Abcam, Cambridge, MA, United States). M1 pro-inflammatory macrophages were identified using mouse anti-inducible nitric oxide synthase (iNOS) antibody (Abcam, Cambridge, MA, United States) followed by Alexa Fluor® 488 conjugated goat anti-mouse IgG (Invitrogen, CA, United States). M2 anti-inflammatory macrophages were stained by rabbit anti-liver Arginase (Arg1) antibody (Abcam, Cambridge, MA, United States) followed by Alexa Fluor® 555 conjugated donkey anti-rabbit IgG (Invitrogen, Carlsbad, CA, United States). Slides were mounted by prolong gold antifade mount with DAPI (Life Technologies, Grand Island, NY, United States). Slides were imaged using a fluorescence microscope (BZ-X800, Keyence, IL, United States). Positive cells in all slides were counted double blinded in three randomly selected areas by two independent researchers. All the samples were recorded as digital images with 200x magnification using a microscope (BZ-X800, Keyence, Itasca, IL, United States).

**TABLE 1:** The numerical scoring scheme used for the histologic evaluation of fracture healing according to Huo et al. (1991).

Score	Associated finding at fracture site
1	Fibrous tissue
2	Predominantly fibrous tissue with small amount of cartilage
3	Equal mixture of fibrous tissue and cartilaginous tissue
4	Predominantly cartilage with small amount of fibrous tissue
5	Cartilage
6	Predominantly cartilage with small amount of immature bone
7	Equal mixture of cartilage and immature bone
8	Predominantly immature bone with small amount of cartilage
9	Union of fracture by immature bone
10	Union of fracture fragments by mature bone

### Statistical Analysis

Statistical analyses were conducted using GraphPad Prism 7 (GraphPad Software, San Diego, CA). Data are presented as mean ± SD. Student t-tests were to compare the tissue mineral content of non-operated femurs between female and male mice. Two-way ANOVA with Bonferroni’s post hoc test was conducted for the multiple statistical comparisons among groups. The difference was considered significant when the *p*-value was < 0.05.

## Results

### Micro-Computational Tomography of the Non-Operative Femur

To determine whether there were differences between sex, the tissue mineral contents of the non-operated femurs were compared. 16 mice of both sexes were measured for this experiment. There were no statistically significant differences in the tissue mineral content between femurs of the non-operative sides of males vs. females (*p* = 0.506) (Figure 2B).

### Micro-Computational Tomography of the Bone Defect Area on the Operative Side

To assess the degree of bone regeneration in the defect area, µCT analysis was performed after 6 weeks to analyze the size and tissue mineral content of the region of interest (Figure 2C). For both sexes, the Sc + IL-4-MSCs groups displayed increased bone healing and decreased bone defect size (Figure 2F). Three of seven male mice receiving Sc + IL-4-MSCs and one of seven female mice receiving Sc + IL-4-MSCs showed bony bridging on µCT; in contrast, none of the other groups showed bony bridging (Figure 2D). The male Sc + IL-4-MSCs group showed higher tissue mineral content compared to the male Sc-only group (Figure 2E).

### Histological Analysis

**FIGURE 3:**
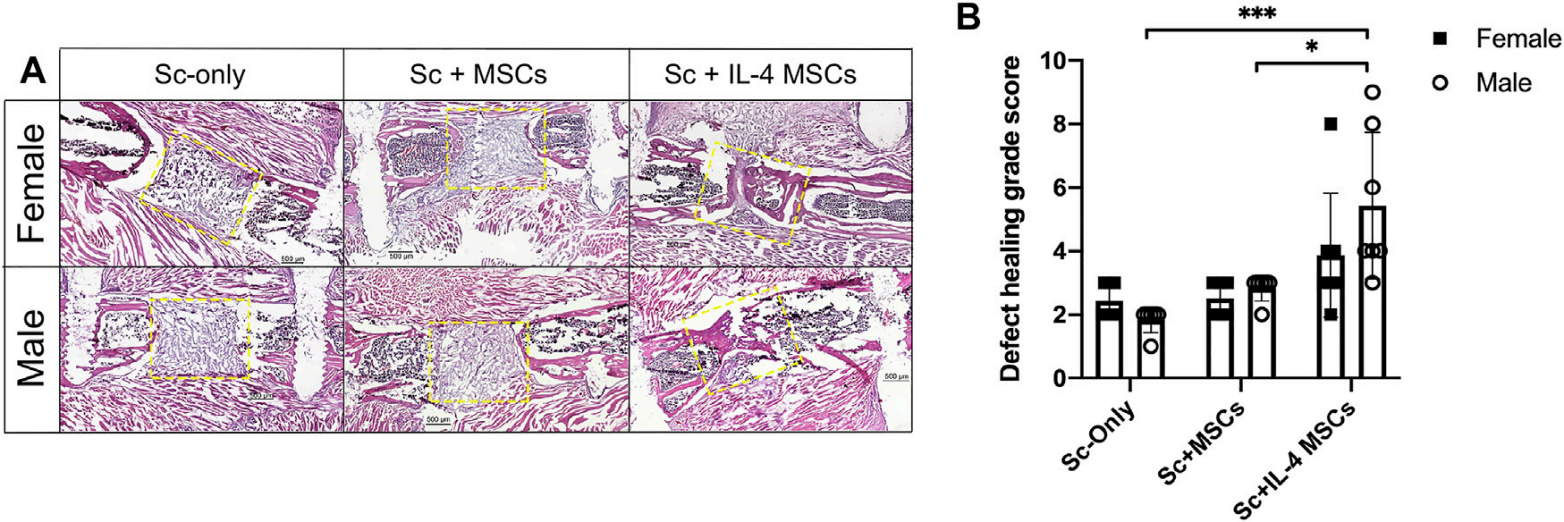
Histology analysis of critical size bone defect healing after 6 weeks in female and male mice. **(A)** H&E stained representative images of the bone defect images (scaffolds ± cells) at 200x magnification. The yellow dotted frames show the original bone defect area. **(B)** Defect healing grade score based on the histological evaluation. *: 0.01 ≤ *p* < 0.05, ***: 0.0001 ≤ *p* < 0.001.

The same trends for the results of µCT were seen with histomorphometric analysis of H&E-stained sections (Figure 3). There was increased bone formed in the periphery of the defect area in Sc + IL-4-MSCs groups of both sexes than in other groups. Three of seven specimens from the male Sc + IL-4-MSCs group and one of seven from the female Sc + IL-4-MSCs group showed bone bridging. None of the mice of the other groups showed bone bridging of the defect. The grade of defect healing quantified by the scoring system of Huo et al., in the male Sc + IL-4-MSCs group (score: 5.4 ± 2.3) was significantly higher compared with the male Sc-only group (score: 1.8 ± 0.4, *p* = 0.0005) and the male Sc + MSC group (score: 2.8 ± 0.4, *p* = 0.022). No significant difference was detected between male and female groups using the same treatment.

### Expression of Alkaline Phosphatase

**FIGURE 4:**
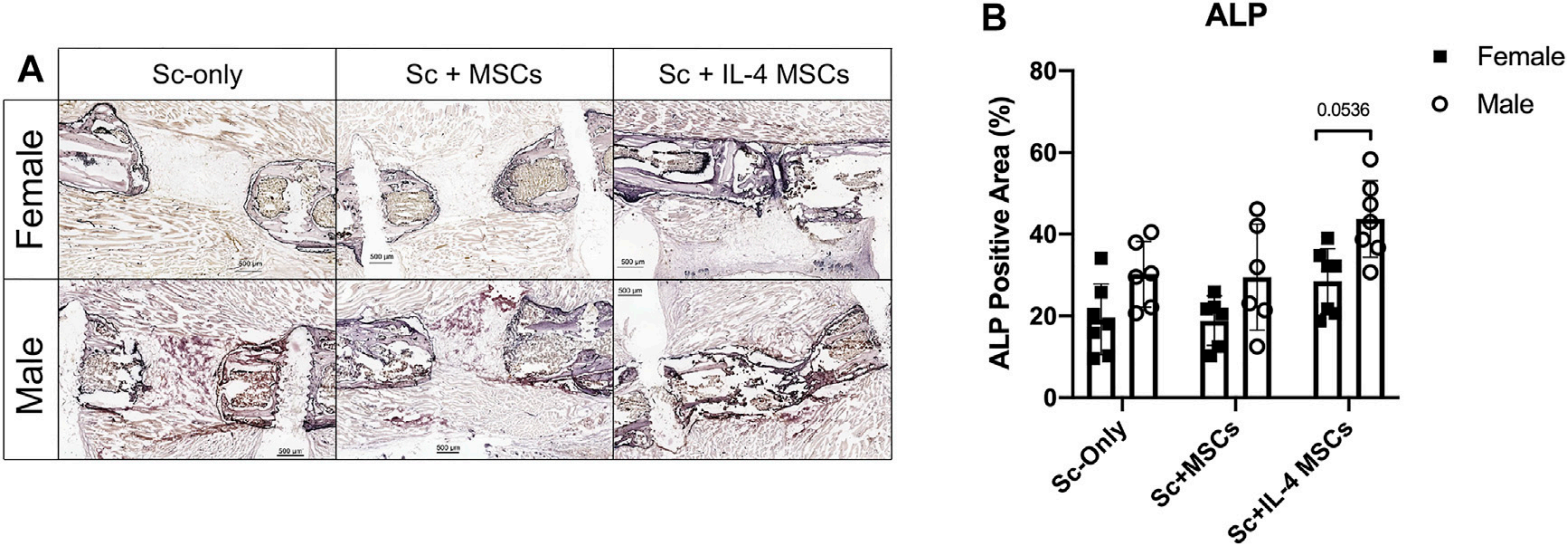
ALP staining and analysis of critical size bone defect healing in female and male mice. **(A)** The ALP-stained bone defects representative images of all groups after 6 weeks with 200 times magnification. **(B)** ALP positive area calculated.

Male groups showed higher ALP positive staining than the female groups for the same treatment, but the values did not reach statistical significance (female vs. male in Sc-only groups: *p* = 0.520, in Sc + MSCs groups: *p* = 0.737). However, the male Sc + IL-4-MSCs group showed a strong trend compared to the female Sc + IL-4-MSCs group (*p* = 0.0536). Comparing the groups in same sex, the Sc + IL-4-MSCs group showed a higher level of ALP positive staining, but this did not reach statistical significance (Figure 4).

### Tartrate Resistant Acid Phosphatase Staining

**FIGURE 5:**
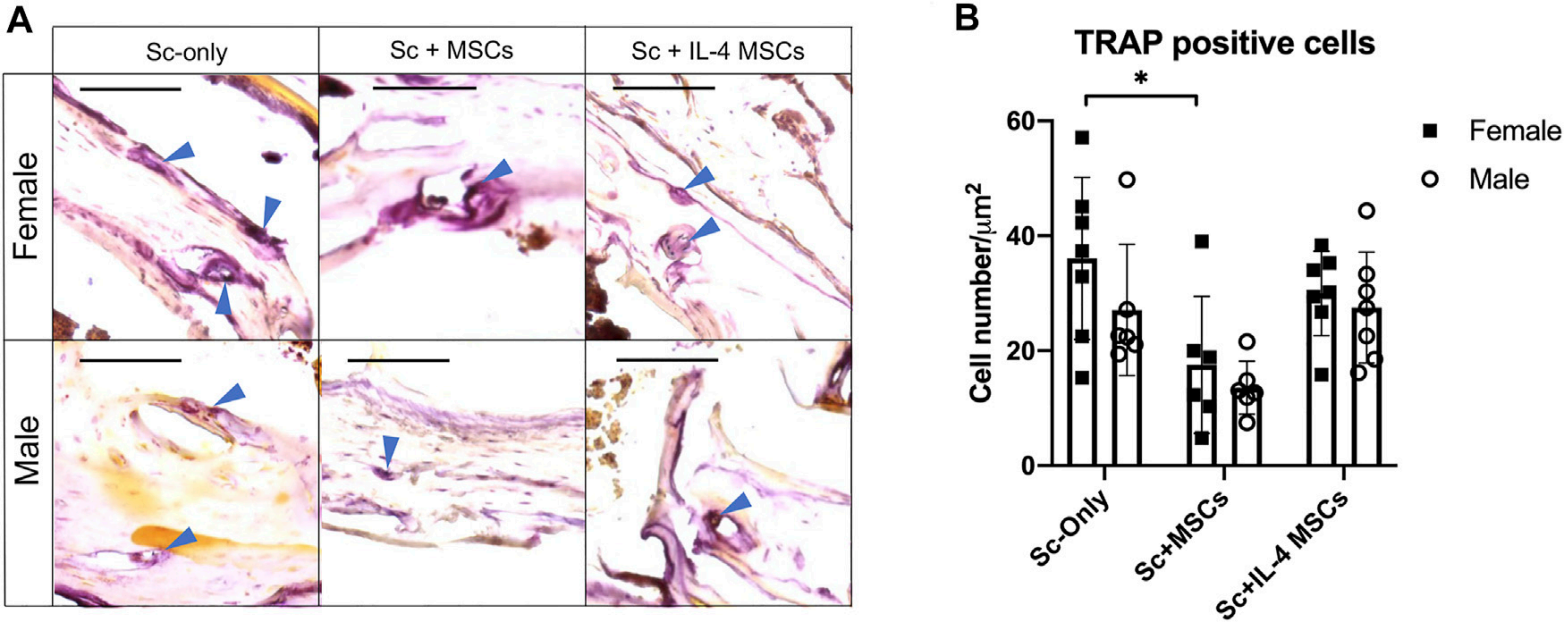
TRAP staining and analysis of critical size bone defect healing after 6 weeks in female and male mice. **(A)** The TRAP-stained bone defects representative images of all groups with 200 times magnification (red arrows showed the TRAP positive cells, bar = 100 µm). **(B)** TRAP positive cell number calculated per µm^2^. *: 0.01 ≤ *p* < 0.05.

For the TRAP staining, the number for female Sc-only group was significantly higher than female Sc + MSCs group: *p* = 0.044). No significant differences were detected among female and male of Sc-only groups, Sc + MSCs groups and Sc + IL-4-MSCs groups (Figure 5).

### Immunohistochemistry for Macrophage Phenotype

**FIGURE 6:**
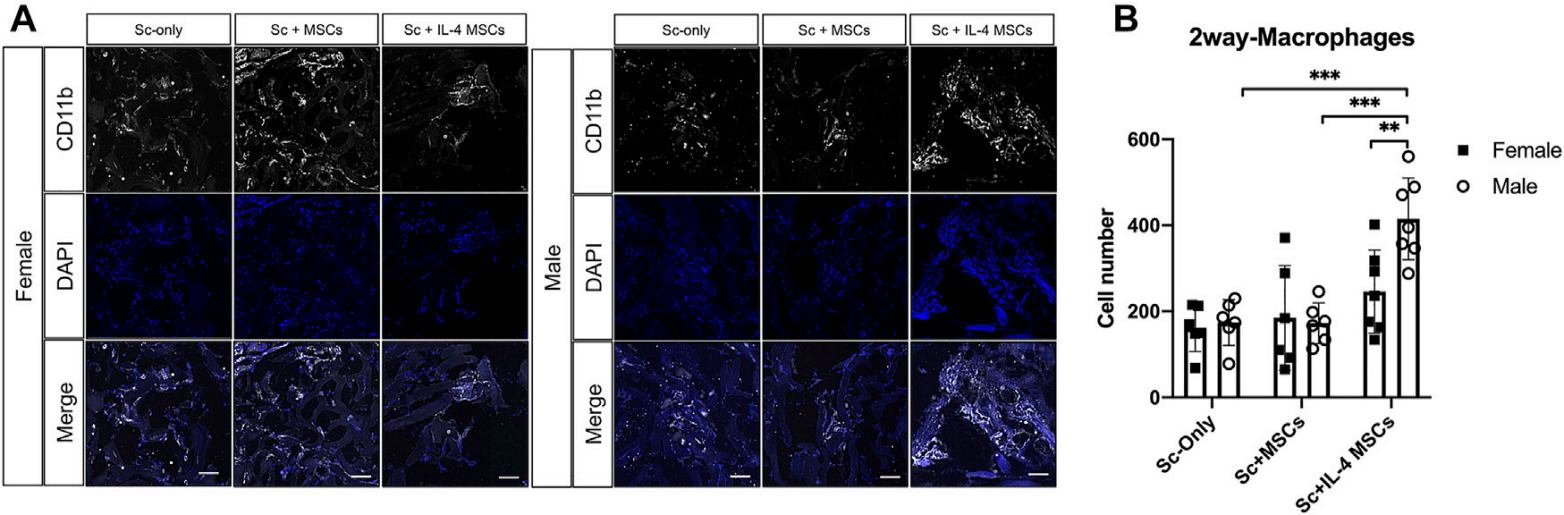
Immunohistochemistry staining and analysis of CD11b/DAPI inside the scaffolds of critical size bone defect healing after 6 weeks in female and male mice. **(A)** Representative images of CD11b/DAPI stained macrophages in all groups. (Blue: DAPI/nucleus; White: CD11b/Macrophage marker. Bar = 100 µm). **(B)** Number of cells counted from the immunohistochemistry images. Female Sc-only, *n* = 6; female Sc + MSCs, *n* = 6; female Sc + IL-4 MSCs, *n* = 7; male Sc-only, *n* = 6; male Sc + MSCs, *n* = 6; male Sc + IL-4 MSCs, *n* = 7. **: 0.001 ≤ *p* < 0.01, ***: 0.0001 ≤ *p* < 0.001, ****: *p* < 0.0001.

**FIGURE 7:**
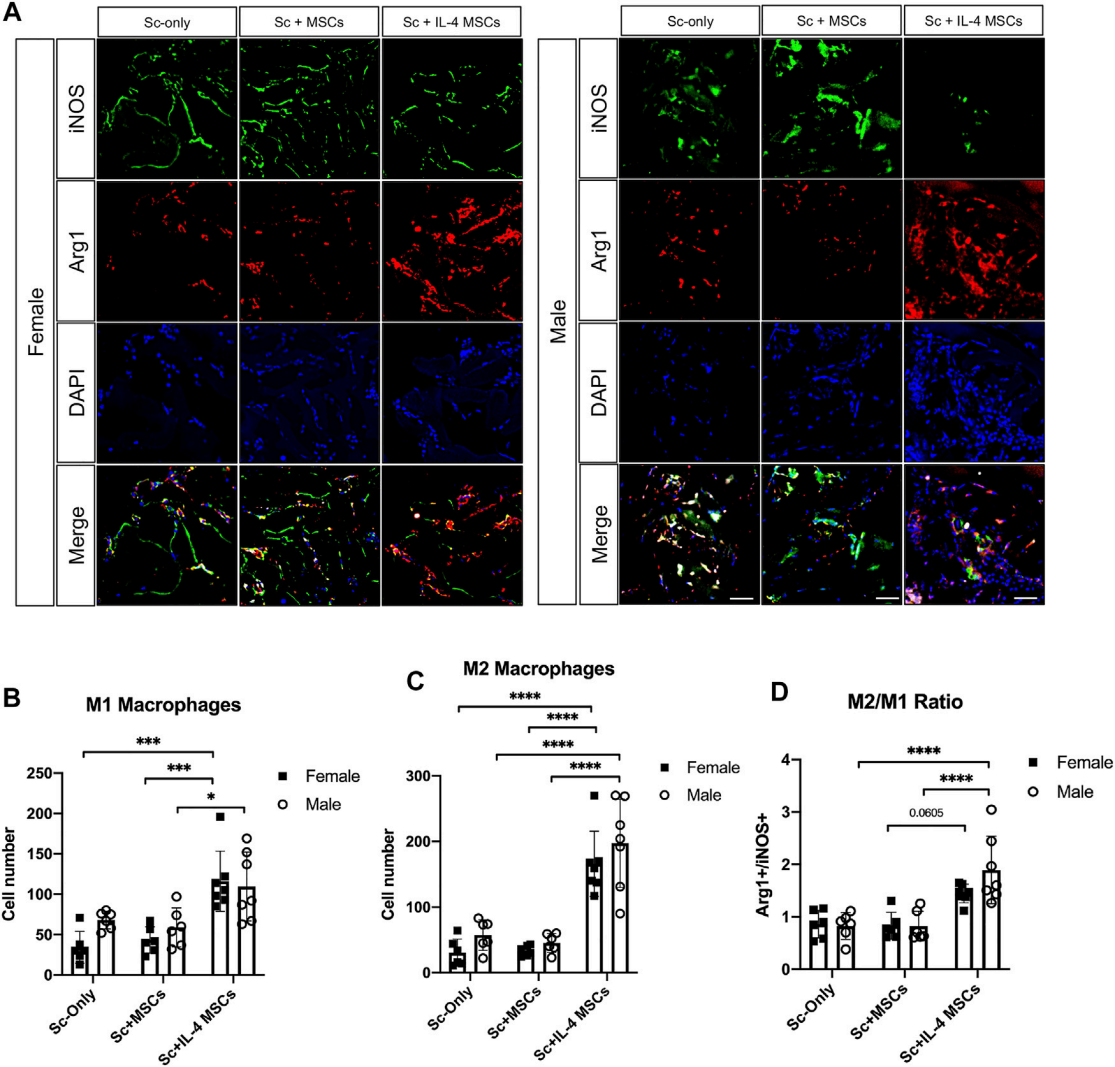
Immunohistochemistry staining and analysis of iNOS/Arg1/DAPI inside the scaffolds of critical size bone defect healing after 6 weeks in female and male mice. **(A)** Representative images of iNOS/Arg1/DAPI stained macrophages in all groups. (Blue: DAPI/nucleus; Green: iNOS/M1 macrophage marker; Red: Arg-1/M2 macrophage marker. Bar = 50 µm). **(B)** Number of cells counted from the immunohistochemistry images. Female Sc-only, *n* = 6; female Sc + MSCs, *n* = 6; female Sc + IL-4 MSCs, *n* = 7; male Sc-only, *n* = 6; male Sc + MSCs, *n* = 6; male Sc + IL-4 MSCs, *n* = 7. *: 0.01 ≤ *p* < 0.05, ***: 0.0001 ≤ *p* < 0.001, ****: *p* < 0.0001.

The number of macrophages (CD11b+/DAPI+) in the male Sc + IL-4-MSCs group was significantly higher than the female Sc + IL-4-MSCs group (*p* = 0.0097). No significant differences were detected among female and male mice in the Sc-only or Sc + MSCs groups. The macrophage number of male Sc + IL-4-MSCs group was significantly increased compared with that in male Sc + MSCs and male Sc-only group (*p* = 0.0002, *p* = 0.0002 respectively). There were no significant differences among the groups of female mice (female Sc-only, female Sc + MSCs, female Sc + IL-4-MSCs) (Figure 6). For the M1 pro-inflammatory macrophage (iNOS+/DAPI+) number, the male Sc + IL-4-MSCs group was significantly higher than the male Sc + MSCs group (*p* = 0.0483); the female Sc + IL-4-MSCs was significantly higher than female Sc + MSCs group (*p* = 0.001) and female Sc-only group (*p* = 0.0002). The number of M2 macrophages in the female Sc + IL-4-MSCs group was significantly increased compared with that in female Sc + MSCs group (*p* < 0.0001) and with that in the female Sc-only group (*p* < 0.0001). M2 macrophage number in male mice showed a similar trend. M2 macrophage number in male Sc + IL-4-MSCs group was significantly increased compared with that in male Sc + MSCs group (*p* < 0.0001) and the male Sc-only group (*p* < 0.0001). The M2/M1 ratio in male Sc + IL-4-MSCs group was significantly higher compared with that in male Sc + MSCs group (*p* < 0.0001) and in the male Sc-only group (*p* < 0.0001). The M2/M1 ratio in female Sc + IL-4-MSCs group showed an increased trend compared with that in male Sc + MSCs group (*p* = 0.0605) and the male Sc-only group (*p* = 0.0913). However, no significant differences of M1 macrophage number, M2 macrophage number or M2/M1 ratio were observed between female and male mice with same implantation treatment (female Sc-only vs. male Sc-only group; female Sc + MSCs vs. male Sc + MSCs group; female Sc + IL-4-MSCs vs. male Sc + IL-4-MSCs group) (Figure 7).

## Discussion

MSCbased bone tissue engineering has been proposed as a potential substitutive intervention to promote bone repair, instead of bulk autologous bone grafting. Recently, we have investigated the healing of bone defects by modulating the immune system (Loi et al., 2016a; Loi et al., 2016b; Goodman et al., 2019; Pajarinen et al., 2019; Maruyama et al., 2020). IL-4 is the most effective anti-inflammatory cytokine that polarizes M1 macrophages to an M2 phenotype (Lin et al., 2017a; Lin et al., 2018; Lin et al., 2019). We previously reported that IL-4 over-expressing MSCs within a μRB scaffold enhanced macrophage migration into the scaffolds and subsequent bone formation to aid in the bridging of a 2 mm a critical size femoral defect in male Balb/c mice, compared to μRB scaffold with or without unmodified MSCs (Ueno et al., 2020). The crosstalk between local MSCs and recruited immune cells at the appropriate time point decreases inflammation and the facilitates bone healing; this enhancement of bone formation by manipulating immune regulation with IL-4 appears to be a generalized phenomenon regardless of sex.

Sex differences have been observed in musculoskeletal diseases. Although the reasons for these differences are complex and largely unknown, one of the possible reasons is the sex-based differences in cells’ response to the local microenvironment (Knewtson et al., 2021). Sex differences have been shown to affect the MSC-based therapeutic potential for bone healing. Katsara et al. found that bone marrow MSCs from male mice had higher osteogenic and adipogenic potential than female mice, without affecting their proliferative abilities (Katsara et al., 2011). Strube et al. reported that female bone marrow contained significantly fewer MSCs, as indicated by lower colony-forming unit (CFU) numbers in femora and tibias using a rat bone defect model (Strube et al., 2009). Male muscle-derived stem cells were more effective than female derived cells in the healing of defects in bone and cartilage (Matsumoto et al., 2008; Meszaros et al., 2012). Mehta et al., demonstrated that male rats showed larger bone callus and advanced healing compared to females using a bone defect model (Mehta et al., 2011). However, little is known concerning the influence of sex on long bone defect healing using MSC-based interventions. Previously, we have compared the gold standard bone-graft (positive control), µRB scaffold only, µRB scaffold with unaltered MSCs and µRB scaffold with IL-4 over-expressing MSCs in the same bone defect model in male mice. The group with bone graft showed enhanced bone formation, however the scaffold only and scaffold with unaltered MSCs groups showed fibrous tissue with virtually no bone formation (Ueno et al., 2020). The group with IL-4 MSCs plus scaffold showed enhanced bone formation. In the current study, we investigated the outcomes of the MSC-based therapy using a µRB scaffold in female and male Balb/c mice subjected to a critical size femoral bone defect.

The current results suggest that IL-4 over-expressing MSCs are an effective way to enhance bone healing in a critical size bone defect model in both female and male mice compared with scaffold only and scaffold with unmodified MSCs. Although the µCT and histological analysis of the bone defect area showed no significant differences between females and males with the same treatment, more detailed analysis revealed some differences based on sex in the mice receiving Sc + IL-4-MSCs. Three of seven male mice and one of seven female mice showed bony bridging after receiving IL-4 over-expressing MSC seeded scaffolds. The ALP staining showed that with the IL-4 over-expressing MSCs scaffold treatment, male mice had a strong trend (*p* = 0.0536, Figure 4B) of increased ALP positive area. Furthermore, the total macrophage number in the male Sc + IL-4-MSCs group was significantly higher than in female mice with the IL-4 over-expressing MSCs intervention; M2 macrophage number, and M2/M1 macrophage ratio also showed that the male Sc + IL-4-MSCs group had a higher level than female mice with the same treatment. These results suggest that male mice had enhanced bone healing with the IL-4 over-expressing MSC seeded scaffolds compared with female mice with the same treatment. These results may be due to sex differences in the properties of the MSCs or the healing process of different sexes, which have been reported by other groups (Strube et al., 2009; Mehta et al., 2011). It should further be taken into account that male animals of the same age have a higher body weight compared to females. In our study, the average body weights of males were higher than females (mean ± SD; male 29.4 ± 1.5 g, female 24.0 ± 1.3 g) which may result in different loading patterns and thus different healing parameters.

Interestingly, the TRAP staining showed that female mice receiving the empty scaffold (female Sc-only) had significantly higher TRAP positive cell number compared with female mice receiving Sc + MSCs (female Sc + MSCs) and higher than female mice receiving Sc + IL-4 MSCs (female Sc + IL-4 MSCs), although no significant difference was detected. Female mice in Sc-only group showed higher TRAP positive cells than male mice receiving the empty scaffold (Figure 5) although no significant differences were found. Similar results have been reported by other researchers using a murine tibial fracture model (Deng et al., 2020). The increased osteoclast numbers (TRAP positive cell number) found in female mice may promote faster remodeling of the fracture calluses.

In the current study, we used IL-4 overexpressing MSCs implanted 3 days after the generation of the critical size bone defect but implanted the scaffold without MSCs and with unmodified MSCs immediately after we created the bone defect. Direct application of anti-inflammatory immunomodulatory cytokines at the critical time can favorably modify the local microenvironment. Administration of IL-4 too early in the bone healing process inhibits the proliferation of human osteoblasts (Frost et al., 2001). Lewis et al. reported that mice overproducing IL-4 had severe osteoporosis primarily due to a marked decrease in osteoblast and ALP activity (Lewis et al., 1993). Our previous *in vitro* study also showed modulation of macrophage phenotype at an appropriate time optimized the proliferation and osteogenic differentiation of MSCs (Loi et al., 2016b). This underscores the concept that the timing of anti-inflammatory cytokine intervention such as IL-4 is critical to facilitating osteogenesis by not interfering with the mandatory pro-inflammatory period of bone healing. Thus, we implanted the scaffolds with IL-4 over-expressing MSCs 3 days after the primary creation of the bone defect.

Autograft is the gold standard for treatment of residual long bone defects however, autologous bone is often limited in quantity and/or quality and can be accompanied by morbidity at the harvest site (Khan et al., 2005). Furthermore, the failure rate of autologous bone grafting to heal a nonunion or other defect ranges from 4 to 25% (Tuchman et al., 2016). Allograft bone is osteoconductive only and may potentially transfer communicable diseases or cause adverse immunological events (Khan et al., 2005). In an effort to find alternative therapies, novel biomaterials were developed. The µRB scaffold used in the current study was reported to enhance the proliferation of stem cells in 3D culture compared with hydrogel scaffold (Han et al., 2016). Cell viability was also promoted in µRB scaffold *in vivo* in a mouse critical-size cranial defect model (Han et al., 2016). In this previous study, BLI results indicated that almost 40% of stem cell survived showed after 6 weeks; this is the same time period after which we implanted the scaffolds in the current study. Together with the addition of MSCs, we showed that the 2-mm defect, which is almost 100% of the femoral diameter in Balb/c mice, was too demanding to bridge the defect after 6 weeks (Ueno et al., 2020). MSC-based therapies using µRB scaffolds are thus promising strategies for healing of bone defects based on the results of this study. However, this treatment was not universally successful in all cases for both sexes.

Limitations to our present study should be recognized. The model represents an acute femoral diaphyseal critical size defect, with harvest at one time point. Ongoing studies using a similar chronic defect will shed further light on these treatments in the face of an established nonunion. The timing of administration of IL-4 over-expressing MSCs was crucial and based on previous *in vitro* and *in vivo* studies. This presents a challenge for clinical translation in which “one-stop” surgical treatment is preferred. Other methods of delivery of the IL-4 payload together with MSCs should be pursued. We also found that the proliferation of female murine MSCs *in vitro* was slower than for males, which is similar to that reported for rat MSCs (Strube et al., 2009). We did not endeavor to detect the specific fate of the implanted MSCs at the end of the current study. A previous report showed an enhancement of cell survival using the µRB scaffold 6 weeks after implantation (Han et al., 2016).

In conclusion, µRB scaffolds with IL-4 over-expressing MSCs improved the healing of critical size femoral diaphyseal bone defects in female and male mice. Male mice had enhanced bone healing using the IL-4 over-expressing MSC seeded scaffolds compared with female mice. These results suggest that sex differences should be considered during the application of MSC-based studies of bone healing.

## Data Availability

The raw data supporting the conclusion of this article will be made available by the authors, without undue reservation.
